# Testosterone-derived estradiol production by male endothelium is robust and dependent on p450 aromatase via estrogen receptor alpha

**DOI:** 10.1186/2193-1801-2-214

**Published:** 2013-05-09

**Authors:** Amparo C Villablanca, Sarada Tetali, Robin Altman, Kenneth F Ng, John C Rutledge

**Affiliations:** Division of Cardiovascular Medicine, University of California, Davis, One Shields Ave., TB 172, Davis, CA 95616-8636 USA; School of Life Sciences, University of Hyderabad, Hyderabad, India

## Abstract

Vascular endothelium expresses both the estrogen receptors (ERs) α and β, and ERα mediates development of early atherosclerosis in male mice. This process is thought to be testosterone-dependent. We hypothesized that male murine aortic endothelium produces robust levels of estradiol by aromatase conversion of testosterone, and that regulation of this process is mediated by the presence of ERs, primarily ERα. Aortic endothelium was isolated from ERα knockout (ERα -/-) and wild-type (ERα +/+) male mice and treated with testosterone or the 5α reduction product dihydrotestosterone (DHT), with or without the P450 aromatase inhibitor anastrazole, or a non-specific estrogen receptor antagonist. Aromatase gene expression and estradiol production were assayed. Treatment with testosterone, but not DHT, caused increased aromatase expression and estradiol production in ERα +/+ endothelium that was attenuated by disruption of ERα in the ERα -/- group. Anastrazole inhibition of aromatase reduced testosterone-induced aromatase expression and estradiol levels in both ERα -/- and ERα +/+ endothelium. Antagonism of both ERs decreased testosterone-induced aromatase expression in both wild-type and knockout groups. The effects of the receptor antagonist on estradiol production differed between the two groups, however, with a reduction in estradiol release from the ERα +/+ cells and complete abolition of estradiol release from the ERα -/- cells. Thus, estradiol production in vascular endothelium from male mice is robust, depends on the aromatic conversion of testosterone and requires functional ERα to achieve maximal levels of estradiol generation. Local vascular production of aromatase-mediated estradiol in response to circulating testosterone may affect ERα-dependent mechanisms to increase susceptibility to early atheroma formation in male mice. This pathway may have important therapeutic relevance for reducing the risk of atherosclerotic cardiovascular disease in human males.

**Electronic supplementary material:**

The online version of this article (doi:10.1186/2193-1801-2-214) contains supplementary material, which is available to authorized users.

## Introduction

Atherosclerotic cardiovascular disease (ASCVD) remains the leading cause of mortality for both men and women in Western societies. However, disparity exists between the incidence of cardiovascular disease in men and women of similar age, as well as between menopausal and pre-menopausal women (Kannel et al.^1976^). Although the mechanisms behind these discrepancies are yet poorly understood, the observations suggest the gender disparities in development of ASCVD stem in part from gender-related differences in sex hormones, primarily estrogen and testosterone.

Historically, much emphasis has been placed on the atheroprotective effects of estrogens in females. This notion of atheroprotective estrogen has been assumed to include males, but accumulating evidence indicates that this simple paradigm does not accurately reflect the complexity of hormonal regulation of vascular disease. Sex hormones exert gender-specific effects, derived from both variations in the levels of sex hormones and from gender-related patterns of hormone and receptor tissue distribution leading to gender-specific responses (Vitale et al.^2009^;Villablanca et al.^2004^;Villablanca et al.^2009^).

Estrogen production depends in large part on the enzymatic conversion of testosterone to estradiol by aromatase, a member of the cytochrome P450 family, which is expressed in the vasculature (Villablanca et al.^2004^;Murakami et al.^2001^;Nathan et al.^2001^). Estrogen levels generated by aromatase activity appear to play a detrimental cardiovascular role in both female and male animal and human models. Aromatase-deficient female mice demonstrated enhanced cardiac mechanical function in an acute ischemia/reperfusion model (Bell et al.^2011^). Work from our lab suggested the importance of estrogen receptor α in mediating early atherogenesis in male mice (Villablanca et al.^2004^). In humans, circulating levels of endogenous estradiol in apparently healthy, middle-aged men positively correlate with carotid artery intima-media thickness (Tivesten et al.^2006^). Additionally, Sudhir and colleagues noted increased susceptibility to early ASCVD in one male individual expressing a mutation of the estrogen receptor (Sudhir et al.^1997^). The interplay between circulating estrogen levels, aromatase-derived production of estrogen, and the downstream effects of estrogen receptor signaling appears to significantly affect the health of the vasculature in males.

In particular, estrogen receptor signaling plays important roles in modulating both genomic and non-genomic pathways that influence inflammatory status and other cellular mechanisms connected with atherosclerotic injury (Villablanca et al.^2010^). The physiological effects of estrogen are mediated by multiple known mechanisms, including two classical receptors located in the cytosol that translocate to the nucleus and act as transcription factors upon ligand binding (ERα and ERβ, encoded by the genes *ESR1* and *ESR2*, respectively). In addition, non-genomic signaling mechanisms include plasma membrane-associated estrogen receptors α and β, and an intracellular G-protein coupled receptor localized to the endoplasmic reticulum (gpER, also known as GPR30) (Meyer et al.^2009^;Meyer & Barton^2009^). Particular attention has been paid to the relative contributions of ERα and β to ASCVD pathophysiology. Vascular tissues in both males and females express ERα and β, although the precise gender differences in expression and distribution of these receptors among various cell types and vascular beds are still unclear (Mendelsohn & Karas^2005^).

Our prior work demonstrated that ERα-mediated increased susceptibility to development of early atherosclerotic lesions in male mice may function in a testosterone-dependent fashion (Villablanca et al.^2004^) via local vascular production of estradiol, as it was not dependent on circulating levels of lipids or testosterone, suggesting local vascular effects. In order to directly test this mechanism, we developed a unique study system whereby we isolated and cultured aortic endothelium from ERα deficient and sufficient male mice. The ERα knockout mouse provides a convenient model to further investigate the mechanisms by which testosterone affects vascular tissues (Couse & Korach^1999^) and the precise involvement of ERα. We sought to establish the extent of local vascular production of estradiol in an *in vivo* model of male vessels where the endothelial contribution can be defined, and to investigate whether the response to this vascular estradiol pool is mediated by estrogen receptors, principally ERα. Because our prior work identified a role for the ERα in vascular atherosclerotic pathology in male mice, we utilized male mice with disruption of ERα and pharmacologic manipulation of hormone status to tease out the interactions of direct effects of testosterone on vascular endothelium, an issue which has not been addressed systematically in the literature.

## Methods

### Animals

ERα knockout (ERαKO) mice, also known as ERαNeo knockout mice, were used in this study. Heterozygous animals were obtained from Dr. Dennis Lubahn (University of Missouri) and mated to yield progeny deficient in the full-length ERα (ERα −/−) and wild-type littermate controls having intact ERα (ERα +/+). ERα −/− animals are deficient in the full-length wild-type ERα protein, and their reproductive function is abolished. However, the disrupting neo sequence used to generate the knockout results in an alternative variant of ERα protein, which retains residual low level estradiol binding activity but lacks the specificity to be from either ERα or ERβ, and animals are thus insensitive to estrogen (Couse et al.^1995^;Kudwa & Rissman^2003^). Mice were housed under standard temperature conditions with a 12-hour light/12-hour dark cycle in a humidity-controlled, dedicated pathogen-free barrier facility at the University of California, Davis. Experiments were performed in compliance with NIH Guidelines and in accordance with protocols approved by the University of California, Davis Animal Care and Use Committee. Tail DNA from progeny of heterozygous matings was obtained using DNeasy spin columns (Qiagen, Valencia, CA) per the manufacturer’s instructions, and PCR amplification was used to distinguish homozygous mutants from heterozygotes and from wild-type animals. Custom primers were designed by Sigma Genosys (The Woodlands, TX). Primer sequences to determine the presence of the targeted ERα gene in homozygous mutants (a 649-bp fragment only) and the wild-type gene in normal animals (a 239-bp fragment only) were as follows: ERα −/− (KO) forward 5’-TGAATGAACTGCAGGACGAG-3’ and reverse 5’-AATATCACGGGTAGCCAACG-3’; ERα +/+ (WT) forward 5’-CTACGGCCAGTCGGGCAT-3’ and reverse 5’-AGACCTGTAGAAGGCGGGAG-3’.

### Endothelial cell harvest and culture

ERα +/+ and ERα −/− littermate male mice at 4 months of age were anesthetized with Nembutal (0.6% ip) and placed on a heating pad to keep them warm. The aortic arch and thoracic aorta were exposed and visualized through a ventral midline incision, and cleaned of adherent fat. The aorta was retrograde perfused with sterile PBS, pH 7.4, and then with EBM Basal Medium Phenol Red Free media (Lonza, Basel, Switzerland). A segment of the thoracic aorta was excised and cut longitudinally, taking care not to injure the endothelial layer, and used for endothelial cell cultures. A summary of methods used for culturing endothelial cells from the above segments is given below.

Mouse aortic explants were placed in tissue culture dishes coated with Matrigel (BD Biosciences, San Jose, CA) and EBM Phenol Red Free media supplemented with a SingleQuot kit (Lonza, Basel, Switzerland) and 10% HyClone defined fetal bovine serum (Thermo Fisher Scientific, Pittsburgh, PA). Segments were placed in the dishes such that the endothelial layer was in contact with the Matrigel. Cultures were maintained at 37°C in a humidified atmosphere with 5% CO_2_. Explants were removed after the cells migrated out from them, and the remaining cells were fed three times weekly with supplemented EBM Phenol Red Free media (growth media) for 4–6 weeks until confluent. Confluent cells were subcultured at a 1:4 split ratio onto uncoated 6-well plates, and used for experiments at P3 at 80-90% confluency.

### Immunohistochemistry

In order to verify the endothelial nature of cells isolated from the mice, immunohistochemical characterization was first performed, demonstrating that the cells were endothelial. Immunostaining for endothelial-specific von Willebrand factor (vWF) was accomplished with a rat anti-vWF antibody (1:50 dilution, Dako Cytomation, Glostrup, Denmark). Cells were seeded on laminin-coated coverslips (Sigma-Aldrich, St. Louis, MO) and fixed using 1% paraformaldehyde. The cells were incubated first with primary vWF antibody, then with biotinylated secondary antibody, and finally with avidin-biotin horseradish peroxidase. Staining was visualized with 3,3′-diaminobenzidine (Vector Laboratories, Burlingame, CA), followed by counterstaining with 10% Mayer’s hematoxylin. The cells were then ethanol and xylene dehydrated and permanently mounted in non-aqueous mounting medium, VectaMount (Vector Laboratories, Burlingame, CA). To confirm that there was no contamination with smooth muscle cells, staining with a rat anti-mouse smooth muscle α-actin antibody was performed (Dako Cytomation, Glostrup, Denmark) as previously described (Martin-McNulty et al.^2007^). Cultured endothelial cells stained positively for vWF and did not show any contamination from smooth muscle following actin staining (data not shown).

### Experimental treatments

Prior to experiments, cells were rendered quiescent by incubation overnight with 2% HyClone charcoal-treated fetal bovine serum (Thermo Fisher Scientific, Pittsburgh, PA). The cells were then incubated for 48 hours at 37°C with media, testosterone, 5α-dihydrotestosterone (DHT), or vehicle control (0.1% ethanol), with or without anastrazole (AK Scientific, Inc., Union City, CA) or ICI-182,780 (Tocris Bioscience, Bristol, United Kingdom), a selective estrogen receptor down-regulator.

### RNA and quantitative real-time PCR

Following experimental treatments, culture media was removed, TRI Reagent (Molecular Research Company, Cincinnati, OH) added directly to the culture dishes, and cells collected by scraping. Total RNA was obtained using TRI Reagent according to the manufacturer’s instructions. Preparation of cDNA was accomplished according to the manufacturer’s instructions for SuperScript II RNase H^-^ Reverse Transcriptase (Invitrogen, Carlsbad, CA) using 4 ug total RNA.

Sample cDNAs were diluted 1:10 and analyzed in duplicate using SYBR Green I Master Mix (Applied Biosystems, Foster City, CA). D-glyceraldehyde-3-phosphate dehydrogenase (GAPDH) primers were designed using Primer Express (Applied Biosystems, Foster City, CA) and were as follows: sense 5'-GCAACAGGGTGGTGGACCT-3' and antisense 5'-GGATAGGGCCTCTCTTGCTCA-3'. Primers for P450 aromatase were commercially obtained (SABiosciences, Valencia, CA). Forward and reverse primers were added at a concentration of 4 uM (P450 aromatase) and 2 uM (GAPDH) for each reaction well. Detection of gene transcript levels was performed using the GeneAmp 7900 HT system (Applied Biosystems, Foster City, CA). The thermal cycling program was as follows: 1 second at 50°C, then thermal activation for 10 min at 95°C and 40 cycles of PCR (melting for 15 seconds at 95°C, followed by annealing/extension for 1 minute at 60°C). Gene expression levels were normalized to GAPDH levels for each sample and expressed as percent change compared to media control. PCR products were separated by electrophoresis on a 2% agarose gel to verify amplification product size.

### Estradiol ELISA assay

After the experimental treatments, the conditioned media from the cell treatments were collected and briefly spun to sediment the dead cells, if any. Supernatants were immediately frozen at −80°C until further use. The concentration of estradiol in the conditioned media (supernatant) was assayed using an Estradiol Enzyme Immunoassay (EIA) kit (Cayman Chemical, Ann Arbor, MI) according to the manufacturer’s instructions. Each experimental treatment was performed in duplicate and the resulting supernatants were assayed in duplicate. Sonicated cell lysates were also tested, and no estradiol was detected in the lysates.

### Statistical analyses

Experimental treatments were performed in duplicate and assayed in duplicate for gene expression or estradiol release. All values for estradiol production and P450 aromatase expression were expressed as means ± standard error of the mean (SEM). Comparisons between study groups were made using Student’s *t*-test for independent samples (two-tailed), and ANOVA, using Microsoft Office 2000 Excel software for PC (Microsoft, Redmond, WA) and SigmaStat version 2.03 (Systat Software, Inc., San Jose, CA). Genotype (ERα −/− versus ERα +/+) and hormone status (testosterone, anastrazole, ICI antagonist) were the grouping variables. Correlations between parameters were analyzed using simple linear regression. A *p*-value of less than 0.05 denoted statistical significance.

## Results

### Disruption of ERα attenuates P450 aromatase expression and abolishes estradiol release in testosterone-treated aortic endothelial cells from male mice

**Figure 1:**
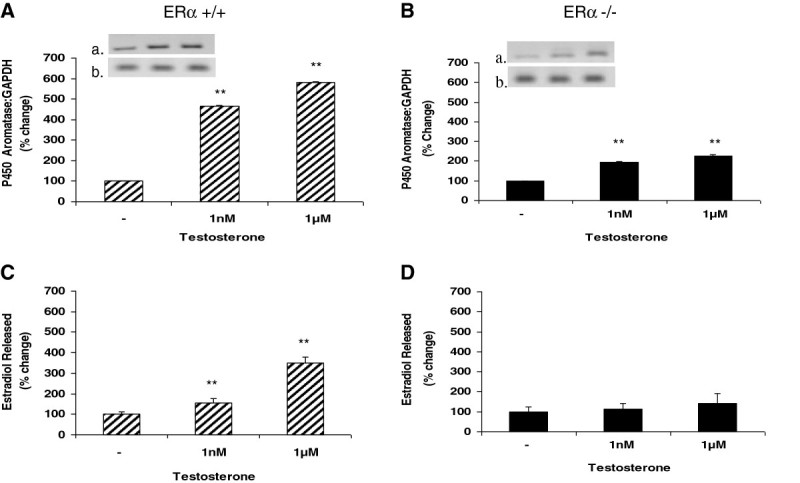
**Testosterone treatment upregulates P450 aromatase expression in murine ERα +/+ and ERα −/− male aortic endothelial cells, and increases estradiol release from ERα +/+ cells.** Aortic endothelial cells isolated from male ERα +/+ and ERα −/− mice were treated with testosterone (1 nM or 1 uM) for 48 hours. Following treatment, total RNA was isolated and used for qRT-PCR analysis of expression of the P450 aromatase gene. Gene expression levels were normalized to GAPDH and expressed as percent change compared to control (panels **A** and **B**). To ascertain the amplification and integrity of the resulting qRT-PCR products, the products were separated on a 2% agarose gel (inset, top panel: P450 aromatase and bottom panel: GAPDH). Cell supernatants were collected following treatment and used for ELISA analysis of estradiol release (panels **C** and **D**). Results are expressed as means ± SEM. ** *p* < 0.01 compared to vehicle control.

We first sought to determine whether and how direct application of testosterone to aortic endothelial cells isolated from ERα +/+ male mice affects estradiol release and expression of P450 aromatase. As shown in Figure 1A and C, testosterone treatment increased P450 aromatase expression and increased estradiol release from the ERα +/+ endothelium in a dose-dependent manner. Treatment with the higher dose of testosterone (1 uM) caused increases in aromatase expression and estradiol production of 580% and 350% of control, respectively. Disruption of the ERα accomplished by use of the ERα −/− mouse resulted in attenuation of the increase in levels of P450 aromatase seen in response to testosterone treatment (Figure 1B). Although P450 aromatase still increased in a dose-dependent manner in the ERα −/− cells, this increase was reduced by two- to three-fold at each dose of testosterone compared to the cells isolated from ERα +/+ male mice. In addition, the ERα −/− condition resulted in near complete abolishment of estradiol production (Figure 1D).

### Reduction in P450 aromatase expression and estradiol release in response to treatment with DHT is unchanged following disruption of ERα in male murine aortic endothelial cells

**Figure 2:**
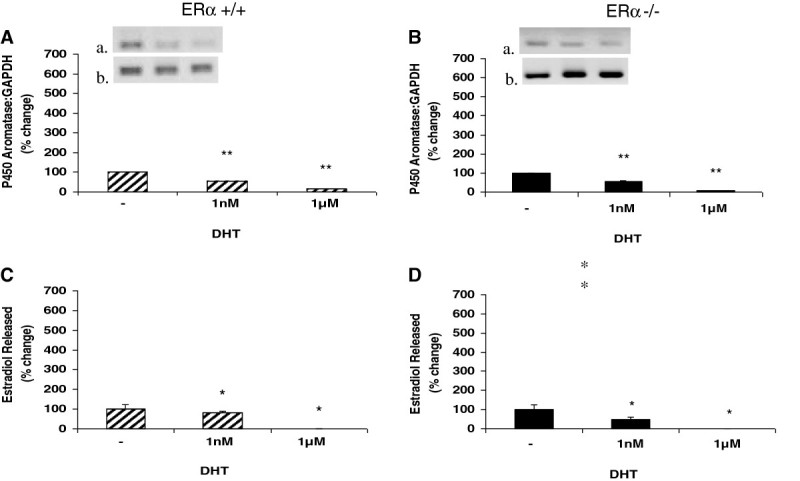
**Dihydrotestosterone (DHT) treatment down regulates P450 aromatase expression and protein levels, and reduces estradiol production from murine ERα +/+ and ERα −/− male aortic endothelial cells.** Aortic endothelial cells isolated from male ERα +/+ and ERα −/− mice were treated with DHT (1 nM or 1 uM) for 48 hours. Following treatment, total RNA was isolated and used for qRT-PCR analysis of expression of the P450 aromatase gene. Gene expression levels were normalized to GAPDH and expressed as percent change compared to control (panels **A** and **B**). To ascertain the amplification and integrity of the resulting qRT-PCR products, the products were separated on a 2% agarose gel (inset, top panel: P450 aromatase and bottom panel: GAPDH). Cell supernatants were collected following treatment and used for ELISA analysis of estradiol release (panels C and D). Results are expressed as means ± SEM. ** *p* < 0.01 and * *p* < 0.05 compared to vehicle control.

Testosterone can be further metabolized to either 5α-dihydrotestosterone (DHT) by the reductive action of the cytochrome P450 enzyme 5α-reductase, or converted to 17β-estradiol by P450 aromatase. DHT is a more potent form of testosterone and has greater androgen receptor binding affinity than testosterone. We treated male murine aortic endothelial cells with DHT to determine whether the observed effects of testosterone on aromatase expression and estradiol release are specific to testosterone itself, or mediated by estradiol generated downstream from testosterone by the actions of aromatase. As shown in Figure 2, increasing concentrations of DHT resulted in decreased aromatase expression and decreased levels of secreted estradiol, and the response to DHT was similar in both the ERα −/− and ERα +/+ endothelial cells. At the highest concentration of DHT (1 uM), secreted estradiol levels were completely abolished (Figure 2C and D), while aromatase expression was reduced to less than 20% of control values for both ERα +/+ and ERα −/− groups (Figure 2A and B).

### P450 aromatase inhibition reduces testosterone-induced P450 aromatase expression and abolishes testosterone-induced estradiol release from aortic endothelial cells from both ERα wild-type and ERα knockout male mice

**Figure 3:**
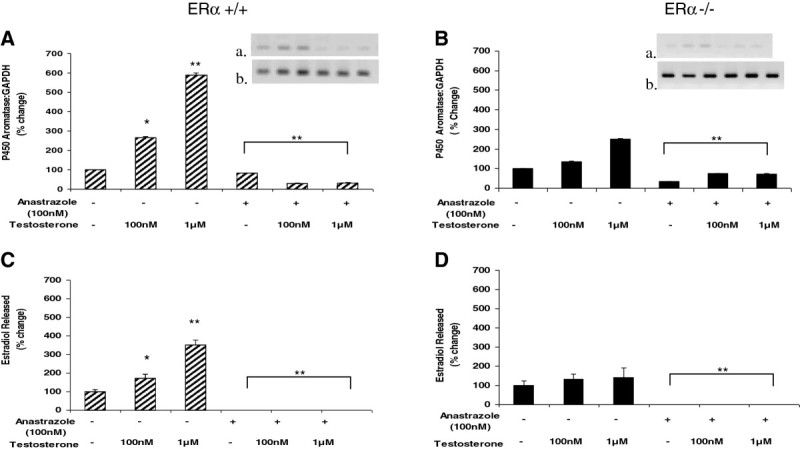
**The P450 aromatase inhibitor, anastrazole, reduces testosterone-induced P450 aromatase expression and estradiol production in murine ERα +/+ and ERα −/− male aortic endothelial cells.** Aortic endothelial cells isolated from male ERα +/+ and ERα −/− mice were treated with testosterone (100 nM or 1 uM) with or without 100 nM anastrazole for 48 hours. Following treatment, total RNA was isolated and used for qRT-PCR analysis of expression of the P450 aromatase gene. Gene expression levels were normalized to GAPDH and expressed as percent change compared to control (panels **A** and **B**). To ascertain the amplification and integrity of the resulting qRT-PCR products, the products were separated on a 2% agarose gel (inset, top panel: P450 aromatase and bottom panel: GAPDH). Cell supernatants were collected following treatment and used for ELISA analysis of estradiol release (panels **C** and **D**). Results are expressed as means ± SEM. ** *p* < 0.01 and * *p* < 0.05 compared to vehicle control.

To further define the role of testosterone-derived estradiol in the mechanisms of estradiol release from aortic endothelium, we then co-incubated isolated cells with testosterone and an aromatase inhibitor, anastrazole (Figure 3). Endothelial cells derived from ERα +/+ male animals demonstrated reduced aromatase expression and no estradiol secretion in response to treatment with both testosterone and anastrazole (Figure 3A and C), compared to the dose-dependent increases in aromatase and estradiol described in response to testosterone alone. A similar trend was observed for the ERα −/− cells, where co-incubation with testosterone and anastrazole caused a reduction in aromatase expression and complete abolishment of estradiol release. Although testosterone treatment alone generated much lower levels of aromatase expression and estradiol release from ERα −/− endothelial cells compared to ERα +/+ cells, the reduction in levels of aromatase expression following anastrazole treatment (30-80% compared to testosterone alone) were not significantly different between the ERα −/− and ERα +/+ groups. There was also no estradiol production in endothelium from ERα −/− and ERα +/+ male mice following anastrazole.

### Testosterone-induced P450 aromatase expression and estradiol release from ERα wild-type and ERα knockout male aortic endothelial cells are attenuated by estrogen receptor antagonism

**Figure 4:**
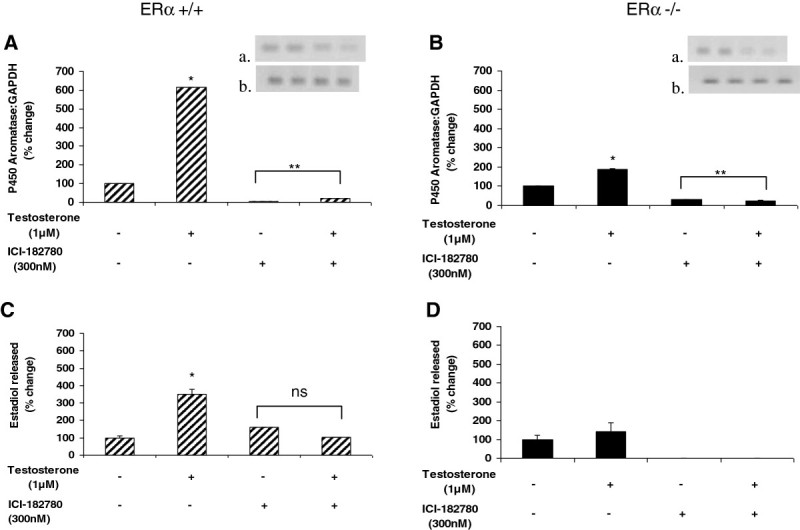
**Antagonism of both estrogen receptors α and β attenuates testosterone-induced P450 aromatase expression and estradiol release from murine ERα +/+ and ERα −/− male aortic endothelial cells.** Aortic endothelial cells isolated from male ERα +/+ and ERα −/− mice were treated with testosterone (1 uM) with or without 300 nM ICI-182,780 (selective estrogen receptor down-regulator that primarily down-regulates ERα, and to a lesser extent ERβ) for 48 hours. Following treatment, total RNA was isolated and used for qRT-PCR analysis of expression of the P450 aromatase gene. Gene expression levels were normalized to GAPDH and expressed as percent change compared to control (panels **A** and **B**). To ascertain the amplification and integrity of the resulting qRT-PCR products, the products were separated on a 2% agarose gel (inset, top panel: P450 aromatase and bottom panel: GAPDH). Cell supernatants were collected following treatment and used for ELISA analysis of estradiol release (panels **C** and **D**). Results are expressed as means ± SEM. ** *p* < 0.01 and * *p* < 0.05 compared to vehicle control; ns = not significant.

As our murine model addressed the contribution of the ERα using the knockout model, but did not address the contribution of the ERβ, we assessed the contribution of each receptor and investigated how blocking both receptors would influence the effects of testosterone on aortic endothelial estrogen production. The estrogen receptor antagonist ICI-182,780 was used to block both ERα and ERβ. ICI-182,780 primarily down-regulates ERα, and to a lesser extent ERβ (Pike et al.^2001^). As shown in Figure 4, antagonism of estrogen receptors by ICI-182,780 reduced aromatase expression in the presence and absence of testosterone in both ERα +/+ and ER −/− animals. In ERα +/+ cells, the antagonist alone reduced aromatase expression to 3% of control, while in the presence of testosterone, the antagonist reduced aromatase expression to 21% of control (Figure 4A). In the ERα −/− group, the antagonist alone reduced aromatase expression to 29% of control, while the antagonist plus testosterone reduced expression to 25% of control (Figure 4B). Estrogen receptor inhibition had no effect on estradiol release compared to control conditions in endothelium from ERα +/+ mice, but decreased testosterone-induced estradiol to baseline values (Figure 4C). In contrast, pharmacological blockade of estrogen receptors in ERα −/− mice completely abolished estradiol release both with and without testosterone stimulation.

## Discussion

Although estrogen’s effects on the vasculature are generally accepted to be atheroprotective in females, there is considerable gender-specific variation regarding sex hormone effects as they relate to cardiovascular disease risk and sex. To further investigate the role of estrogen in the male vasculature, we sought to determine whether and how estrogen is locally produced by male endothelium, the level of production, and the estrogen receptor (principally α) dependence utilizing endothelial cells derived from aortic explants from ERα wild-type (+/+) and knockout (−/−) male mice. In this unique study system, we have characterized the level of endothelial testosterone-enhanced estradiol production and identified an estrogen receptor-dependent operative mechanism. Our results demonstrate that male aortic endothelial cells do indeed produce relatively large quantities of estrogen, and this process proceeds through the enzymatic conversion of testosterone by P450 aromatase. In addition, ERα plays a primary role in this pathway by contributing to increased aromatase expression and estradiol release, with a lesser contribution from ERβ.

Testosterone is the primary sex steroid present in males, although there are appreciable quantities of estrogen present in the circulation. In males, the enzymatic conversion of testosterone by P450 aromatase throughout the body provides the major source of estrogen. Previous studies including our own have demonstrated the presence of aromatase in various tissues, including the vascular endothelium (Villablanca et al.^2004^;Murakami et al.^2001^;Nathan et al.^2001^). Estrogen at the level of the vasculature is customarily thought of as atheroprotective due to its ability to exert anti-inflammatory and anti-atherogenic effects. Epidemiological and observational studies originally noted decreased incidence of cardiovascular disease in pre-menopausal women compared to age-matched men (Kannel et al.^1976^;Regitz-Zagrosek^2006^;Störk et al.^2004^). Additional studies in female mice demonstrated similar protective effects of estrogen in models of cardiovascular injury (Brouchet et al.^2001^;Pare et al.^2002^;Dean et al.^2005^). Some cell culture models also provide evidence of the anti-inflammatory and anti-atherogenic roles of estrogen (Mori et al.^2004^;Florian & Magder^2008^), but these do not provide a conclusive understanding, as other cell experiments demonstrate the opposite effects of estrogen, including upregulation of inflammatory markers in endothelial cells co-stimulated with tumor necrosis factor-α (Zhang et al.2002a;Zhang et al.2002b). The vast majority of evidence supporting estrogen’s vasculoprotective role has been generated in female models, and the conflicting cellular results from experiments where the sex of the donor is often unknown suggest the importance of independently investigating the male response before assuming similarity between males and females.

To begin addressing these sex-specific differences, we first sought to determine whether murine male aortic endothelium could produce estradiol under basal conditions and in the presence of testosterone. Under control conditions very little estradiol was generated; however, testosterone stimulation caused a large increase in the production of estradiol (to over 400 pg/ml) that was completely abolished by co-incubation with an aromatase inhibitor, anastrazole, suggesting that local vascular production of estrogen is dependent on the activity of aromatase in male mice. Our findings correlate well with previous studies demonstrating the necessity of aromatase for estrogen-mediated effects in male tissues (Sun et al.^2007^;Sierra-Ramirez et al.^2004^;Kimura et al.^2003^). Further, when we treated cells with 5α-dihydrotestosterone (DHT), a form of testosterone that cannot be converted to estradiol by aromatase, we did not observe any estradiol production by aortic endothelial cells derived from either the ERα +/+ or ERα −/− animals. This lends support to our conclusion that vascular production of estradiol in male mice depends on the P450 aromatase-driven conversion of testosterone.

In response to testosterone stimulation, gene expression of aromatase increased in a dose-dependent manner in both the ERα +/+ and ERα −/− endothelial cells, but was reduced in the ERα −/− group compared to the ERα +/+ cells. The partial ERα gene present in the ERα −/− mice may be responsible for this persistent partial upregulation of aromatase in ERα −/− mice in the presence of testosterone. Our studies with the selective estrogen receptor (alpha greater than beta) down-regulator, ICI-182,780, also support this interpretation. Testosterone-induced estradiol release from ERα −/− endothelium also was significantly attenuated compared to ERα +/+ endothelium, suggesting involvement of the ERα in the aromatase-mediated mechanism of estradiol release as well. Others have shown similar intricate connections between ERα, aromatase, and estradiol in other biological systems. In hippocampal neurons for example, aromatase-mediated estrogen production appears to be involved in an auto/paracrine feedback mechanism to regulate ERα and β expression (Prange-Kiel et al.^2003^). Additionally, Kinoshita and Chen demonstrated the necessity of ERα in estrogen-mediated aromatase activity in human breast cancer cells (Kinoshita & Chen^2003^).

Pharmacological inhibition of both classical ERs by a selective estrogen receptor down-regulator helped to further define the relative contributions of ERα and β to estrogen-induced responses in the aortic endothelium of male mice. We observed attenuation of testosterone-induced estradiol production in the ERα +/+ endothelium in the presence of the antagonist, and interestingly, complete abolishment of estradiol production in response to testosterone stimulation in the ERα −/− cells with the antagonist. We interpret these results to indicate that the presence of functional ERα helps maintain baseline levels of aromatase available to generate estradiol. Antagonism of both ERα and β in the ERα +/+ group reduced testosterone-stimulated estradiol levels to control values, but the presence of the intact ERα appears to have previously primed the cells such that they were able to produce baseline levels of estrogen even in the presence of the antagonist. However, the absence of ERα seems to leave the cells deficient in their ability to produce baseline levels of estrogen, and inhibiting the remaining ERβ completely prevents estradiol release as observed in the ERα −/− group. Based on our results, we estimate that approximately two thirds of the estradiol produced in response to testosterone stimulation in male wild-type mice is due to the presence of ERα, and the remaining one third is due to the presence of ERβ. However, the possible role for ERβ requires future work with endothelium from ERβ knockout mice and/or specific ERβ inhibitors.

The results reported here demonstrate the importance of ERα and P450 aromatase in mediating testosterone-induced estradiol release from male murine aortic endothelium. Our previous findings demonstrated increased susceptibility to early atherosclerotic vascular changes in male mice mediated by the ERα and suggested likely dependence on testosterone (Villablanca et al.^2004^). Although this present study was not intended to address atherogenesis, in the context of our prior studies, our findings suggest that the mechanism for early atherogenesis in ERα −/− male mice could indeed be explained by local vascular estrogen production. This proposed mechanism is in contrast to the findings of other groups, such as those demonstrating aromatase-mediated anti-inflammatory effects of testosterone (Mukherjee et al.^2002^). However, it is important to note the female origin of the models used for these studies, which likely display different receptor and response patterns compared to males. Work by Chakrabarti et al. suggests a complicating factor may lie in how the different types of estrogen receptors respond upon estrogen binding, as their results demonstrate that anti-inflammatory estrogen signaling through the gpER in human umbilical vein endothelial cells is complicated by concomitant signaling through ERs α and β serving to attenuate estrogen’s anti-inflammatory response (Chakrabarti & Davidge^2012^). The question necessitates future studies to unravel the complex interactions between the many different estrogen receptors and determine how these interactions differ between males and females.

Although a number of previous studies have demonstrated testosterone-driven regulation of early atherogenic events in vascular endothelium (Nathan et al.^2001^;Mukherjee et al.^2002^), to our knowledge, our report is the first to demonstrate the level of robustness of estradiol production by male mouse endothelium. In addition, we demonstrated that regulation of this process depends not only on the aromatic conversion of testosterone, but also on the presence of a functional ERα. Although earlier work suggested that human endothelium did not express aromatase (Harada et al.^1999^), subsequently P450 aromatase expression was demonstrated in a variety of vascular tissues in humans (Murakami et al.^2001^;Mukherjee et al.^2002^;Dietrich et al.^2011^;Diano et al.^1999^;Sasano et al.^1999^), thereby establishing the biologic relevance of our work in a murine system to the human vasculature. The results of our studies in murine endothelium therefore provide an additional model of investigation regarding gender-specific differences in the hormonal regulation of vascular disease.

The issue of gender differences in endothelium is complex, and the results reported herein raise an interesting question about the physiological relevance of locally-produced estrogen in males. It remains unclear whether estrogen generated at the level of the vascular endothelium contributes deleterious cardiovascular effects in males. It is also unclear whether aromatase activity and testosterone action differ between endothelial vascular beds (e.g., coronary endothelium, aortic endothelium, etc.) and across animal species (e.g., mouse, rat, other) (Sierra-Ramirez et al.^2004^). There are reported differences amongst animal species, sex, and the nature of the vascular endothelium being studied. Further work is clearly needed in this important and interesting area of study to fully unravel the mechanisms underlying the complexity observed by our studies and those of prior investigators. It will be important to pursue future studies aimed at investigating why male endothelium expresses aromatase and produces estrogen, and the significance of these mechanisms as they relate to atheroma formation in males. Our work has direct clinical relevance, as it emphasizes the necessity of exploring gender-specific differences in sex hormone-related effects on vascular health, and suggests ERα-dependent mechanisms as potential therapeutic targets to decrease risk of atherosclerotic cardiovascular disease, not only in females, but also in males.

## Authors’ original submitted files for images

Below are the links to the authors’ original submitted files for images. Authors’ original file for figure 1 Authors’ original file for figure 2 Authors’ original file for figure 3 Authors’ original file for figure 4
