# Supplementary material for: Testosterone-derived estradiol production by male endothelium is robust and dependent on p450 aromatase via estrogen receptor alpha
Source: Springerplus. 2013 May 9;2(1):214. doi: 10.1186/2193-1801-2-214 (PMC3667361; doi:10.1186/2193-1801-2-214)
Supplement: Supplementary file 1 — Authors’ original file for figure 1 [file 40064_2013_289_MOESM1_ESM.pdf]

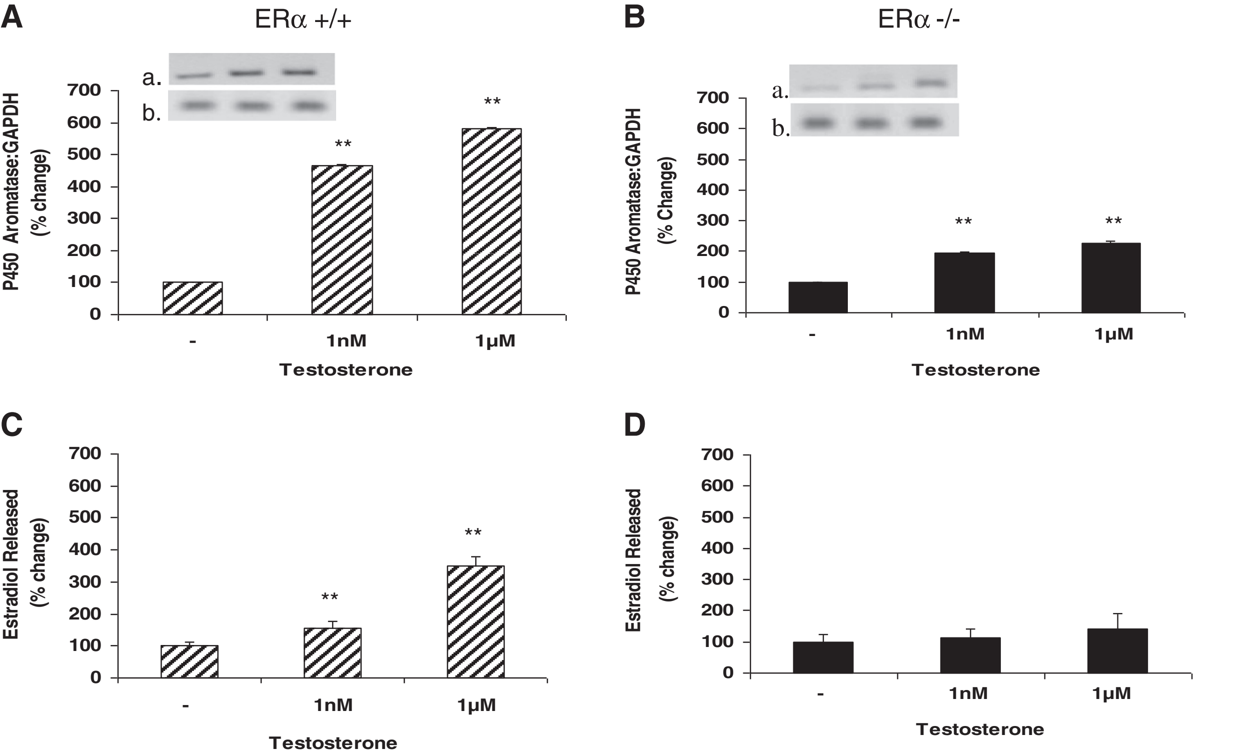

A
ERα +/+
a.
b.
700
600
500
400
300
200
100
0
P450 Aromatase:GAPDH (% change)
**
**
-
1nM
1μM
Testosterone
B
ERα -/-
a.
b.
P450 Aromatase:GAPDH (% Change)
**
**
-
1nM
1μM
Testosterone
C
Estradiol Released (% change)
**
**
-
1nM
1μM
Testosterone
D
Estradiol Released (% change)
-
1nM
1μM
Testosterone
