# Supplementary figures and images for: Testosterone-derived estradiol production by male endothelium is robust and dependent on p450 aromatase via estrogen receptor alpha
Source: Springerplus. 2013 May 9;2(1):214. doi: 10.1186/2193-1801-2-214 (PMC3667361; doi:10.1186/2193-1801-2-214)

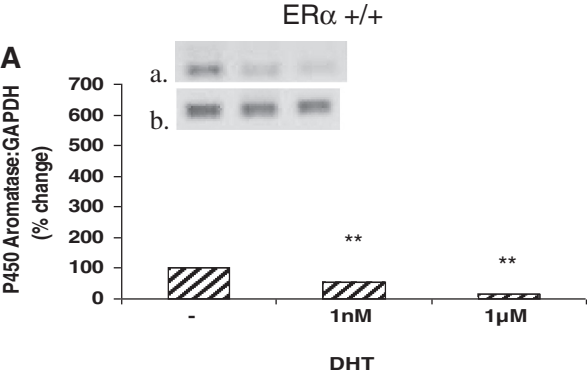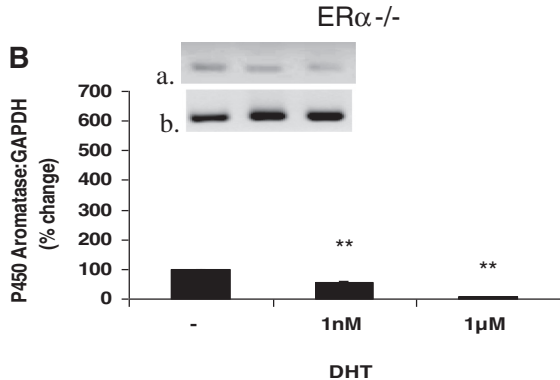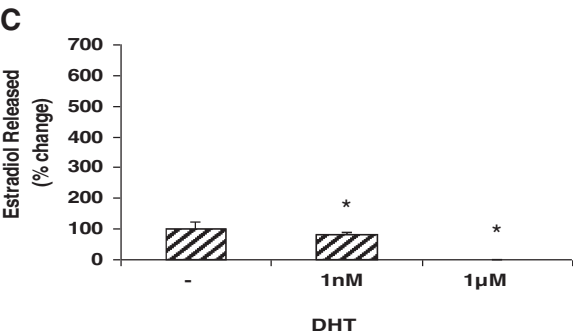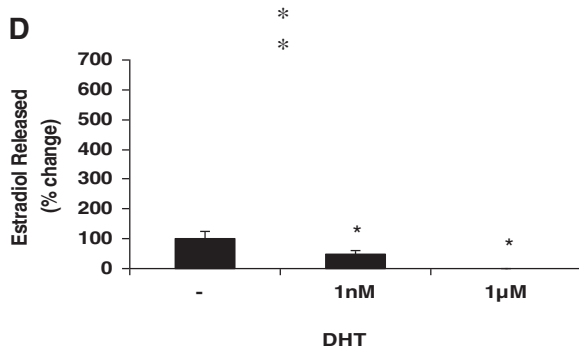

Supplement: Supplementary file 2 — Authors’ original file for figure 2 [file 40064_2013_289_MOESM2_ESM.pdf]

ER $\alpha$  +/+**A**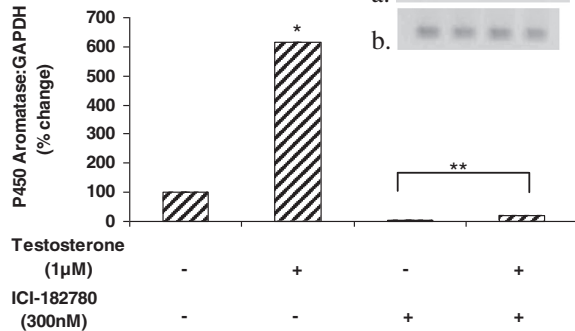ER $\alpha$  -/-**B**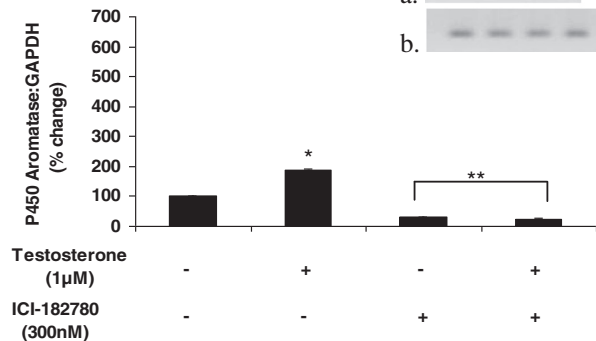**C**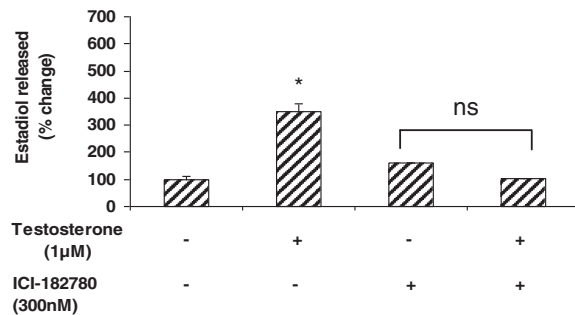**D**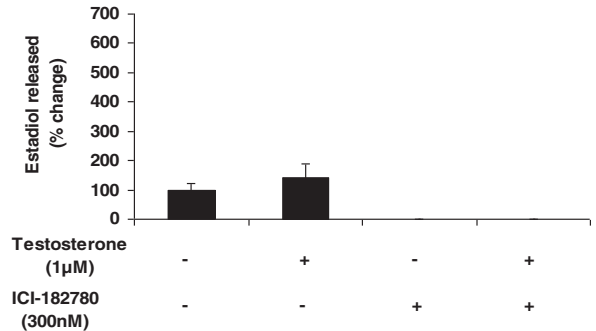

Supplement: Supplementary file 4 — Authors’ original file for figure 4 [file 40064_2013_289_MOESM4_ESM.pdf]
