# Supplementary material for: Testosterone-derived estradiol production by male endothelium is robust and dependent on p450 aromatase via estrogen receptor alpha
Source: Springerplus. 2013 May 9;2(1):214. doi: 10.1186/2193-1801-2-214 (PMC3667361; doi:10.1186/2193-1801-2-214)
Supplement: Supplementary file 3 — Authors’ original file for figure 3 [file 40064_2013_289_MOESM3_ESM.pdf]

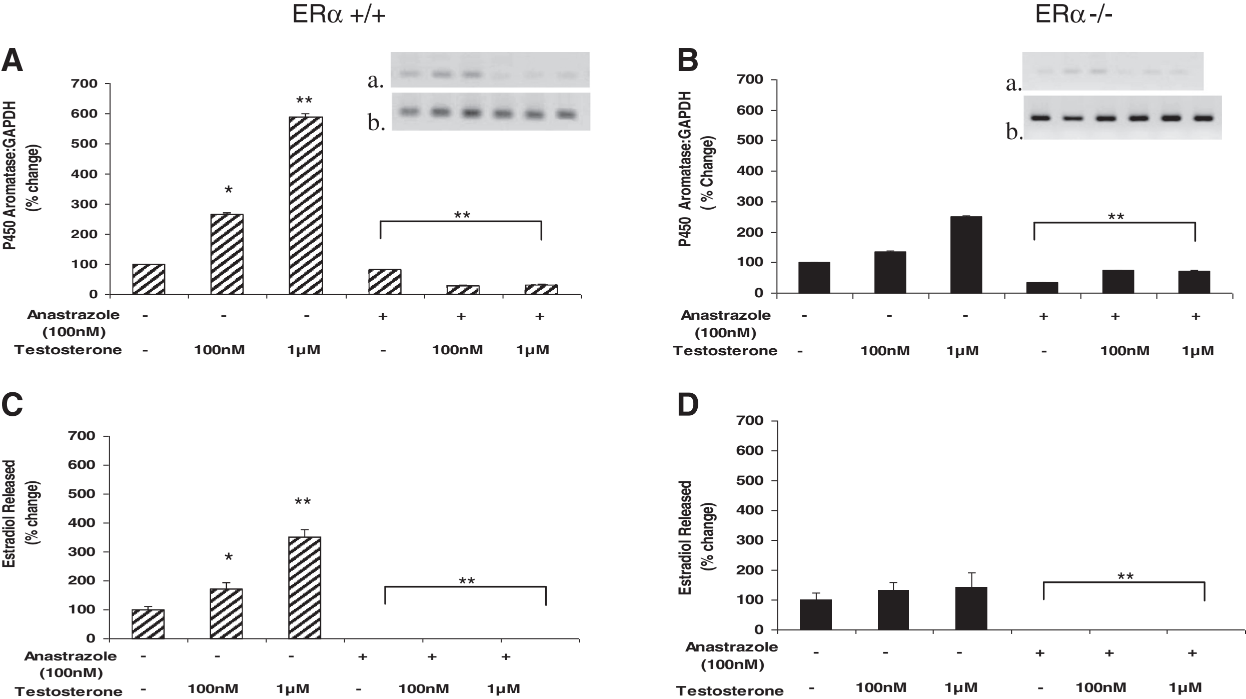
ERα +/+
ERα -/-
A
a.
b.
P450 Aromatase:GAPDH (% change)
700
600
500
400
300
200
100
0
*
**
**
Anastrazole (100nM)
-
-
-
+
+
+
Testosterone
-
100nM
1μM
-
100nM
1μM
B
a.
b.
P450 Aromatase:GAPDH (% Change)
**
C
Estradiol Released (% change)
*
**
**
D
Estradiol Released (% change)
**
